# Impact of Targeted Deletion of the Circadian Clock Gene Bmal1 in Excitatory Forebrain Neurons on Adult Neurogenesis and Olfactory Function

**DOI:** 10.3390/ijms21041394

**Published:** 2020-02-19

**Authors:** Amira A. H. Ali, Federica Tundo-Lavalle, Soha A. Hassan, Martina Pfeffer, Anna Stahr, Charlotte von Gall

**Affiliations:** 1 Institute of Anatomy II, Medical Faculty, Heinrich-Heine-University, Merowinger Platz 1a, 40225 Düsseldorf, Germany; amira.ali@med.uni-duesseldorf.de (A.A.H.A.); Federica.Tundo-Lavalle@med.uni-duesseldorf.de (F.T.-L.); soha.hassan@suezuniv.edu.eg (S.A.H.); Martina.Pfeffer@med.uni-duesseldorf.de (M.P.); stahranna@web.de (A.S.); 2 Zoology Department, Faculty of Science, Suez University, Suez 43111, Egypt

**Keywords:** circadian clock, neurogenesis, oxidative stress, hippocampus

## Abstract

The circadian system is an endogenous timekeeping system that synchronizes physiology and behavior with the 24 h solar day. Mice with total deletion of the core circadian clock gene Bmal1 show circadian arrhythmicity, cognitive deficits, and accelerated age-dependent decline in adult neurogenesis as a consequence of increased oxidative stress. However, it is not yet known if the impaired adult neurogenesis is due to circadian disruption or to loss of the Bmal1 gene function. Therefore, we investigated oxidative stress and adult neurogenesis of the two principle neurogenic niches, the hippocampal subgranular zone and the subventricular zone in mice with a forebrain specific deletion of *Bmal1* (*Bmal1 fKO*), which show regular circadian rhythmicity. Moreover, we analyzed the morphology of the olfactory bulb, as well as olfactory function in *Bmal1 fKO* mice. In *Bmal1 fKO* mice, oxidative stress was increased in subregions of the hippocampus and the olfactory bulb but not in the neurogenic niches. Consistently, adult neurogenesis was not affected in *Bmal1 fKO* mice. Although Reelin expression in the olfactory bulb was higher in *Bmal1 fKO* mice as compared to wildtype mice (*Bmal1 WT)*, the olfactory function was not affected. Taken together, the targeted deletion of *Bmal1* in mouse forebrain neurons is associated with a regional increase in oxidative stress and increased Reelin expression in the olfactory bulb but does not affect adult neurogenesis or olfactory function.

## 1. Introduction

In mammals, the circadian system is the internal timekeeping system that coordinates physiology and behavior with the 24 h solar day. An essential part of this system is the master circadian pacemaker in the hypothalamic suprachiasmatic nucleus (SCN). The phase and period of the circadian pacemaker can be entrained by external environmental cues such as the daily light changes. The SCN contains a neuronal network that provides rhythmic signals to other brain regions and to the entire body. On the cellular level, the SCN neurons and almost all other cells in the body contain cell-autonomous circadian molecular clocks [1]. The core circadian molecular clockwork consists of two interlocked transcription/translation feedback loops (TTFL). The transcriptional activators’ brain and muscle Arnt-like protein1 (BMAL1) and circadian locomotor output cycles (CLOCK) heterodimerize and activate the transcription of other clock genes, e.g., *Period* (*Per*) and Cryptochrome (*Cry*). PER and CRY proteins translocate into the nucleus, heterodimerize and inhibit CLOCK:BMAL1 and, in turn, their own transcription. Also, CLOCK: BMAL1 complex enhances transcription of nuclear receptors, REV-ERBa, and RORa, which control *Bmal1* transcription [2,3,4].

Mice with a conventional targeted deletion of the essential circadian clock gene *Bmal1* (*Bmal1-/-*) show not only a loss of circadian rhythms [5] and an impairment of light entrainment [6], but also other severe impairments, including premature aging and cognitive deficits [7,8]. Moreover, we could show *Bmal1-/-* mice show accelerated age-dependent decline in adult neurogenesis [9] and accelerated migration of neural progenitor cells (NPCs) [10], presumably, as a consequence, of oxidative stress [9]. However, it is still unknown if these effects are due to chronodisruption or to a cell-intrinsic role of *Bmal1*. Previous work showed that conditional deletion of *Bmal1* from Calcium/calmodulin-dependent protein kinase type II subunit alpha (Camk2a) expressing excitatory forebrain neurons (*Bmal1 fKO*), does not affect the SCN clock and the animals have intact circadian rhythms; however, it led to deficits in cognition and memory formation, indicating that *Bmal1* is involved in forebrain neuronal networks [11]. In this study, we addressed the question if the forebrain specific deletion of *Bmal1* affects the neurogenic brain niches and adult neurogenesis.

Adult neurogenesis is the process of continuous generation of newborn neurons and their subsequent integration into the pre-existing circuits [12,13]. This occurs mainly in two forebrain areas in mammals: the subventricular zone (SVZ) of the lateral ventricle (LV) and the dentate gyrus (DG) of the hippocampus. In the DG, adult neurogenesis includes proliferation, migration, and differentiation/maturation of NPCs, and, finally, integration within the hippocampal circuits [14]. Hippocampal neurogenesis influences the encoding of new memories [15] and has an obvious relationship to spatial memory formation [16] and cognition [17].

On the other hand, the SVZ gives rise to NPCs that migrate tangentially along the rostral migratory stream (RMS) for a distance of up to 5 mm to the olfactory bulb (OB) [18]. The RMS is wrapped by glial fibrillary acidic protein-positive (GFAP+) astrocytes that direct the migrating NPCs to the OB [19]. In the OB, the NPCs migrate radially to the cortex. The change from tangential to radial migration of the NPCs to the olfactory cortex involves detachment of NPCs from the chains and is mediated by many factors, including Reelin [20]. Then, the NPCs differentiate into interneurons within the granule cell layer (GCL) and the glomerular layer (GL) and become integrated into the OB neuronal network [21]. These adult-born interneurons play an important role in processing odor information and display a high degree of synaptic plasticity [22].

In this study, we analyzed proliferation, migration, differentiation of NPCs, and morphology of the two main neurogenic niches in the hippocampus and SVZ in *Bmal1 fKO* mice. As conventional deletion of *Bmal1* is associated with high reactive oxygen species (ROS) levels [7] affecting adult neurogenesis [9,10], we analyzed oxidized nucleobase indicative for oxidative stress. Moreover, we analyzed Reelin, which is not only a regulator of migrating neurons [23] but also a marker for olfactory input [24].

## 2. Results

### 2.1. Forebrain Specific Bmal1 Deletion Leads to Subregional Increases in Oxidative Stress but not to Astrocyte Activation

**Figure 1:**
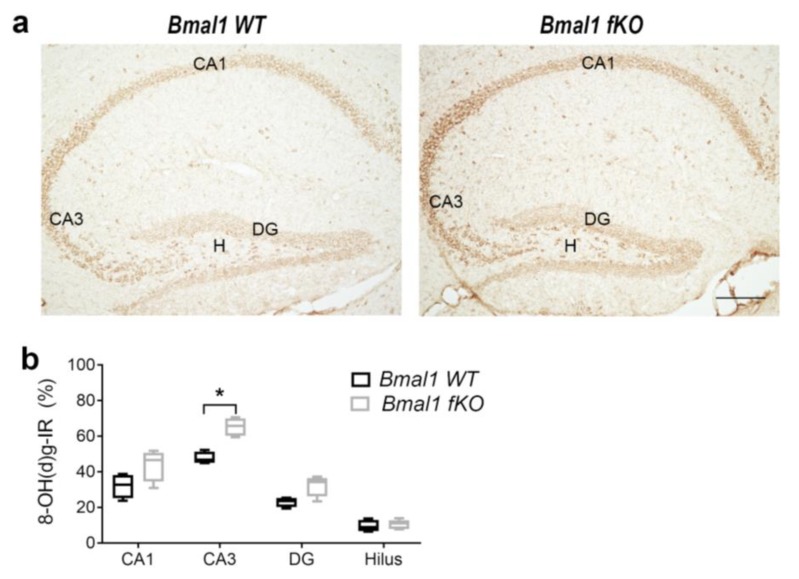
Oxidative stress in the hippocampus of mice with conditional deletion of *Bmal1* from forebrain excitatory neurons (*Bmal1 fKO*) mice. (**a**) Representative photomicrograph of the oxidative stress marker 8-hydroxydeoxyguanosine immunoreaction (8-OH(d)g-IR) in the hippocampus of wildtype mice (*Bmal1 WT*) and *Bmal1 fKO*. Scale bar = 200 µm. (**b**) Quantitative analysis of 8-OH(d)g-IR in hippocampal subregions of *Bmal1 WT* and *Bmal1 fKO* mice. * *p* < 0.05. DG, dentate gyrus, H, hilus, CA, cornu ammonis.

As conventional *Bmal1-/-* mice show impaired adult neurogenesis in the dentate gyrus presumably as a consequence of oxidative stress [9], we analyzed oxidative stress in the conditional *Bmal1 fKo* mice using the oxidative stress marker, 8-hydroxydeoxyguanosine (8-OH(d)g). Perinuclear 8-OH(d)g-immunoreaction (IR) was not significantly different between wildtype mice (*Bmal1 WT*) (*n* = 4) and *Bmal1 fKo* (*n* = 4) mice in the DG (*p* = 0.1), hilus (*p* = 0.4), and CA1 (*p* = 0.1) (Figure 1). However, in the CA3 subregion 8-OH(d)g-IR was significantly higher in *Bmal1 fKO* as compared to *Bmal1 WT* (*p* = 0.03) (Figure 1). This shows a selective subregional increase in oxidative stress upon forebrain-specific neuronal *Bmal1* deletion.

**Figure 2:**
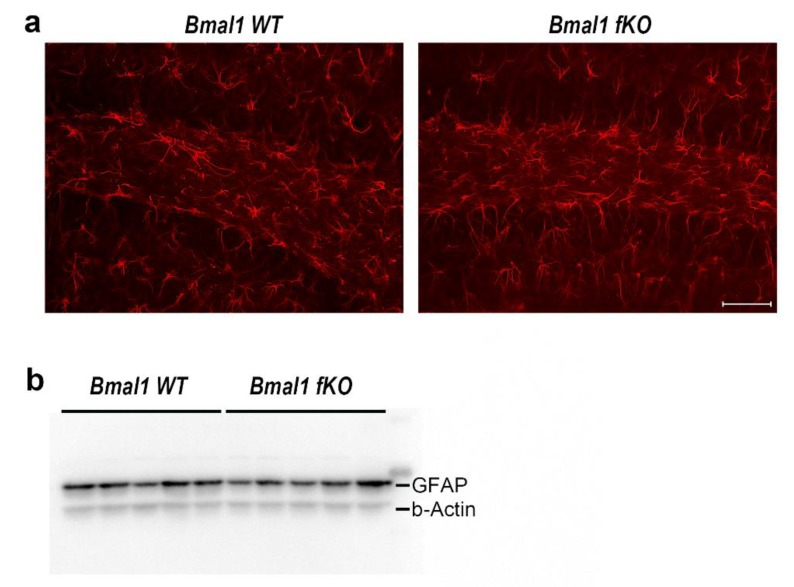
Astrocyte activation in the hippocampus of *Bmal1 fKO* mice. (**a**) Representative immunofluorescence of the astrocytic marker glial fibrillary acidic protein (GFAP) in the dentate gyrus of *Bmal1 WT* and *Bmal1 fKO* mice. Scale bar = 50 µm. (**b**) Immunoblot of GFAP in hippocampus lysates of *Bmal1 WT* and *Bmal1 fKO* mice, *n* = 5 mice per genotype.

Conventional *Bmal1-/-* mice and *NestinCRE^+^;Bmal1^f/f^* mice show increased activation of astrocytes indicated by the increased expression of GFAP [25]. Thus, we analyzed GFAP expression in the hippocampus of *Bmal1 WT* (*n* = 4) and *Bmal1 fKO* (*n* = 4) mice by immunofluorescence and immunoblot. Forebrain specific deletion of *Bmal1* did not lead to the activation of astrocytes in the hippocampus, as GFAP-IR (Figure 2a) and relative GFAP expression (Figure 2b) were not significantly different between *Bmal1 WT* and *Bmal1 fKO* mice (GFAP-IR, *p* = 0.4; relative GFAP-expression, *p* = 0.9).

### 2.2. Forebrain Specific Bmal1 Deletion does not Affect Adult Neurogenesis in the Dentate Gyrus

**Figure 3:**
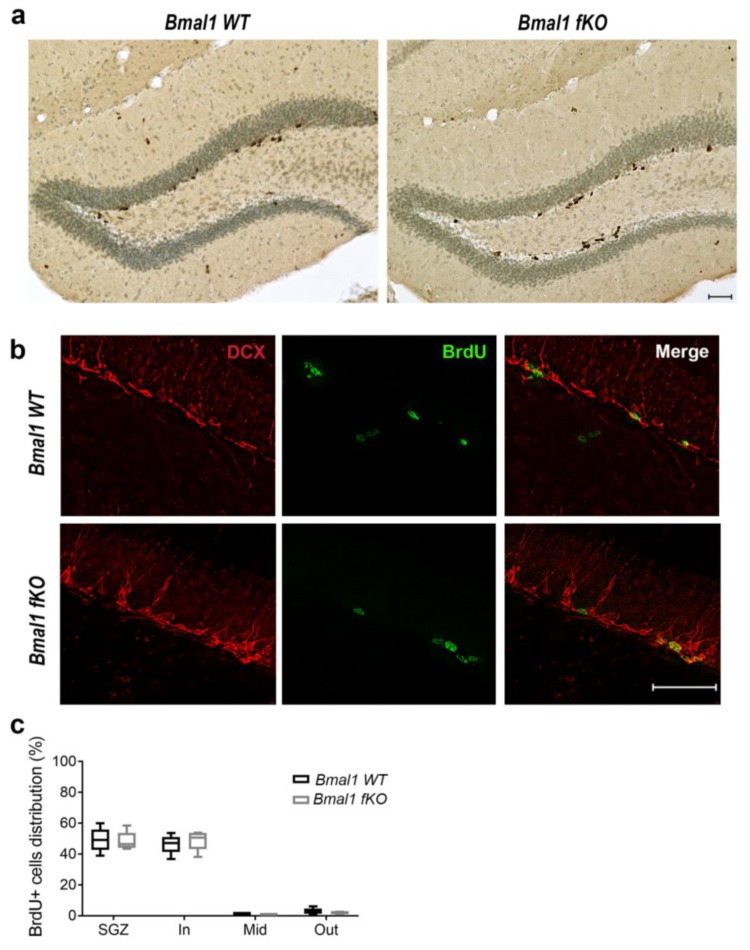
The proliferation and spatial distribution of neural progenitor cells in the dentate gyrus of *Bmal1 fKO* mice. (**a**) Representative photomicrograph of Bromodeoxyuridine-positive (BrdU+) cells (brown) and cresyl violet staining (blue) in the dentate gyrus of *Bmal1 WT* and *Bmal1 fKO* mice. Scale bar = 50 µm. (**b**) Representative immunofluorescence of the marker for proliferating cells BrdU+ (green) and the marker for neuronal precursors/migrating neuroblasts, doublecortin (DCX) (red). (**c**) Quantification of BrdU+ cells within the subgranular zone (SGZ), the inner (In), middle (Mid) or an outer (Out) third of the granular cell layer of the dentate gyrus. *n* = 5 of each genotype.

Proliferation and distribution of NPCs in the dentate gyrus were analyzed by Bromodeoxyuridine (BrdU) assay. One day after the last BrdU injection, BrdU+ cells were arranged in clusters or distributed sporadically, mainly in the subgranular zone (SGZ) of both genotypes (Figure 3a). The number of proliferating BrdU+ cells did not differ between *Bmal1 WT* (3950+/−691) and *Bmal1 fKO* (3132+/−225) mice (*p* = 0.3, *n* = 5 per genotype).

For the further analysis of the proliferating cells, BrdU was co-labeled with the marker for neuronal precursors and migrating neuroblasts, doublecortin (DCX). The percentage of BrdU/DCX-co-labeled cells was not different between *Bmal1 WT* (44.2+/−5.4) and *Bmal1 fKO* (49.6+/−2.7) mice (*p* = 0.6, *n* = 4 per genotype) (Figure 3b). This shows that specific *Bmal1*-deficiency in the forebrain neurons does not affect the proliferation of neuroblasts. Moreover, the subregional spatial distribution of the proliferating BrdU+ cells in the SGZ and the subdivisions of the granular cell layer of the dentate gyrus was not different between the two genotypes (Figure 3c).

**Figure 4:**
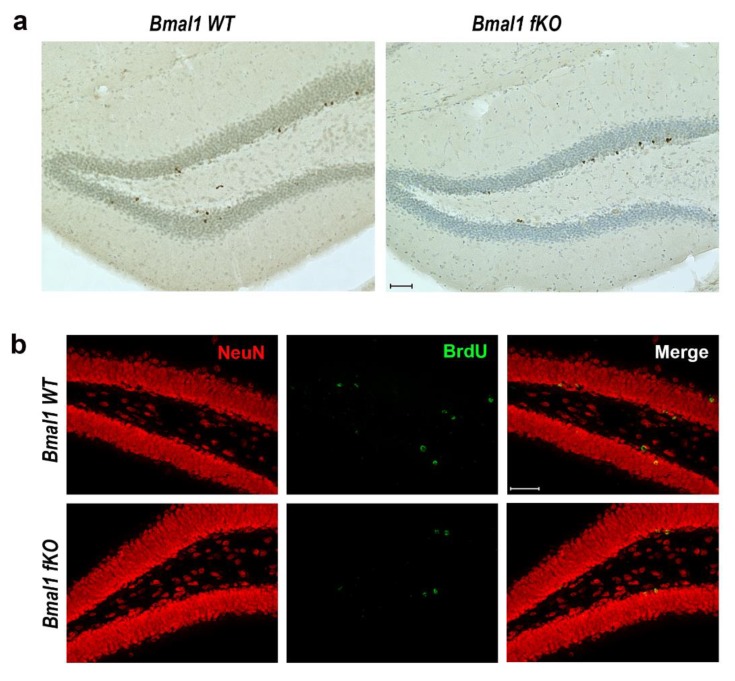
Survival and neural differentiation of proliferating neural progenitor cells in the dentate gyrus of *Bmal1 fKO* mice. (**a**) Representative photomicrograph of BrdU+ cells (brown) and cresyl violet staining (blue) in the dentate gyrus of *Bmal1 WT* and *Bmal1 fKO* mice. Scale bars = 50 µm. (**b**) Representative photomicrograph of BrdU+ (green) and the marker for mature neurons NeuN+ (red) in the dentate gyrus of *Bmal1 WT* and *Bmal1 fKO* mice. Scale bars = 50 µm.

For the analysis of the effect of forebrain specific *Bmal1* deletion on the survival of adult-born cells in the hippocampus, BrdU+ cells were analyzed four weeks after the last BrdU injection. The number of BrdU+ cells in the DG was comparable between *Bmal1 WT* (750+/−117) and *Bmal1 fKO* (715+/−105) mice (*p* = 0.9, *n* = 5 per genotype) (Figure 4a). Thus, forebrain specific *Bmal1* deletion does not affect the survival of adult-born cells. For further analysis of the survival of adult-born mature neurons, the percentage of cells co-labeled for BrdU and neuronal marker NeuN was determined four weeks after the last BrdU injection. In both genotypes, most of the BrdU+ cells expressed NeuN. The percentage of BrdU/NeuN+ cells was comparable between *Bmal1 WT* (81.6+/−4.2) and *Bmal1 fKO* (86.9+/−4.7) mice (*p* = 0.5, *n* = 4 per genotype) (Figure 4b). Consistently, there was no significant difference in the total DG volume between *Bmal1 WT* (0.36+/−0.04 mm^3^) and *Bmal1 fKO* (0.36+/−0.03 mm^3^) mice (*p* = 0.9, *n* = 3 per genotype). Taking these findings together, we presume that the survival and fate decisions of NPCs in the hippocampus were not affected by forebrain specific deletion of *Bmal1*.

### 2.3. Forebrain Specific Bmal1 Deletion Affects Oxidative Stress in the Mitral Cell Layer (MCL) of the Olfactory Bulb (OB)

**Figure 5:**
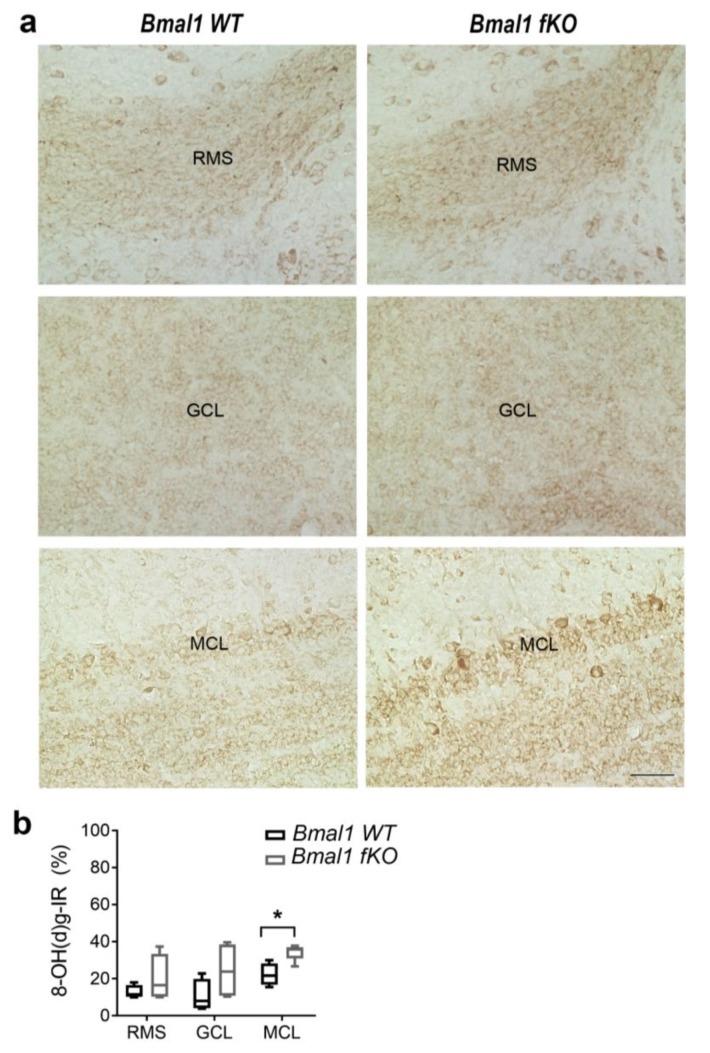
Oxidative stress in the rostral migratory stream and the olfactory bulb of *Bmal1 fKO* mice. (**a**) Representative immunoreaction (IR) of the oxidative stress marker 8-OH(d)g in the rostral migratory stream (RMS), the granular cell layer (GCL) and the mitral layer (MCL) of the olfactory bulb of *Bmal1 WT* and *Bmal1 fKO* mice. Scale bar = 50 µm. (**b**) Quantitative analysis reveals significantly higher 8-OH(d)g-IR in the MCL of *Bmal1KO* as compared to *Bmal1 WT* mice (* *p* < 0.05). *n* = 4 mice of each genotype.

As conventional *Bmal1*-deficiency affects NPC migration in the RMS and the OB presumably, as a consequence of oxidative stress [10], we analyzed 8-OH(d)g-IR in the RMS and the OB of *Bmal1 fKO* mice (*n* = 4 per genotype). 8-OH(d)g-IR was not different in both the RMS (*p* = 0.7) and the GCL (*p* = 0.2) (Figure 5). However, the MCL showed enhanced 8-OH(d)g-IR in *Bmal1 fKO* as compared to *Bmal1 WT* mice (*p* = 0.03) (Figure 5). This is consistent with the impact of forebrain specific *Bmal1*-deletion on oxidative stress levels in selective brain subregions.

### 2.4. Forebrain Specific Bmal1 Deletion does not Affect Adult Neurogenesis in the Subventricular Zone and the Rostral Migratory Stream

**Figure 6:**
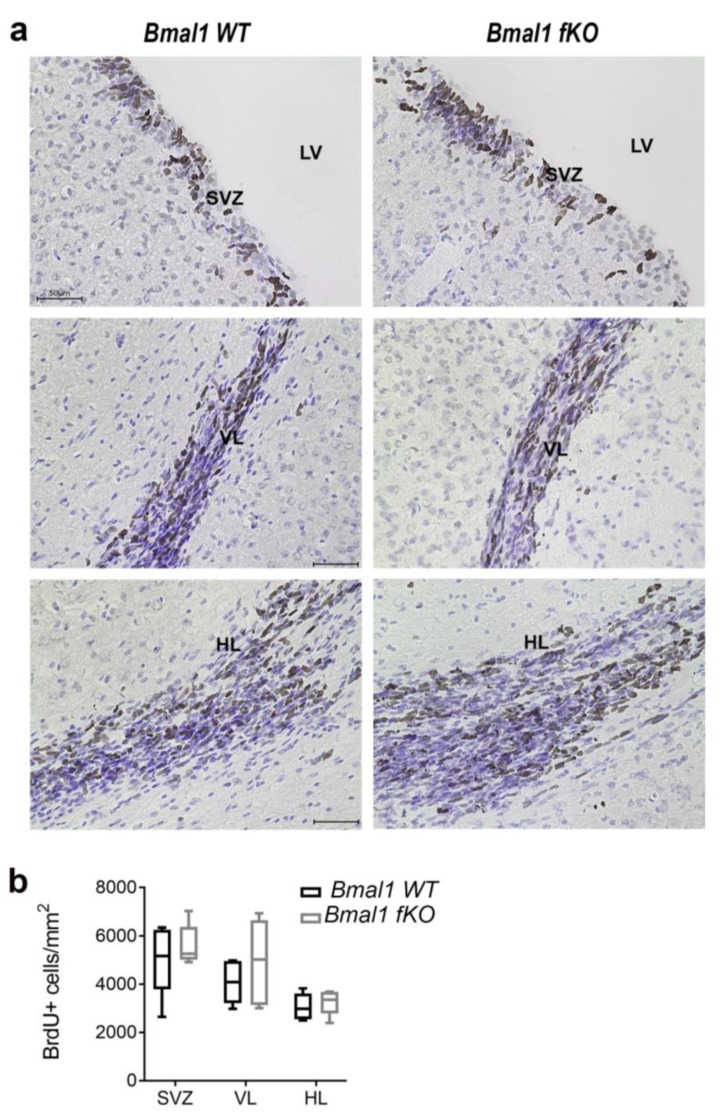
Proliferating cells in the subventricular zone and the rostral migratory stream in *Bmal1 fKO* mice. (**a**) Representative photomicrograph of BrdU+ cells (brown) and cresyl violet staining (blue) in the subventricular zone (SVZ), the vertical limb of the rostral migratory stream (VL), and the horizontal limb of the rostral migratory stream (HL). Scale bars = 50 µm. LV = lateral ventricle. (**b**) Quantitative analysis of BrdU+ cells in the SVZ, the VL, and the HL. *n* = 5 mice per genotype.

The proliferation of NPCs in the SVZ and the RMS was analyzed by BrdU assay one day after the last BrdU injection (*n* = 5 per genotype). The NPC proliferation in the neurogenic niches of the SVZ and the RMS was not affected by forebrain specific deletion of *Bmal1*, as there was no significant difference in the number of BrdU+ cells between *Bmal1 WT* and *Bmal1 fKO* mice in the SVZ (*p* = 0.5), in the vertical limb of the RMS (*p* = 0.4) or in the horizontal limb of the RMS (*p* = 0.6) (Figure 6).

### 2.5. Forebrain Specific Bmal1 Deletion does not Affect Migration of NPCs to the OB

**Figure 7:**
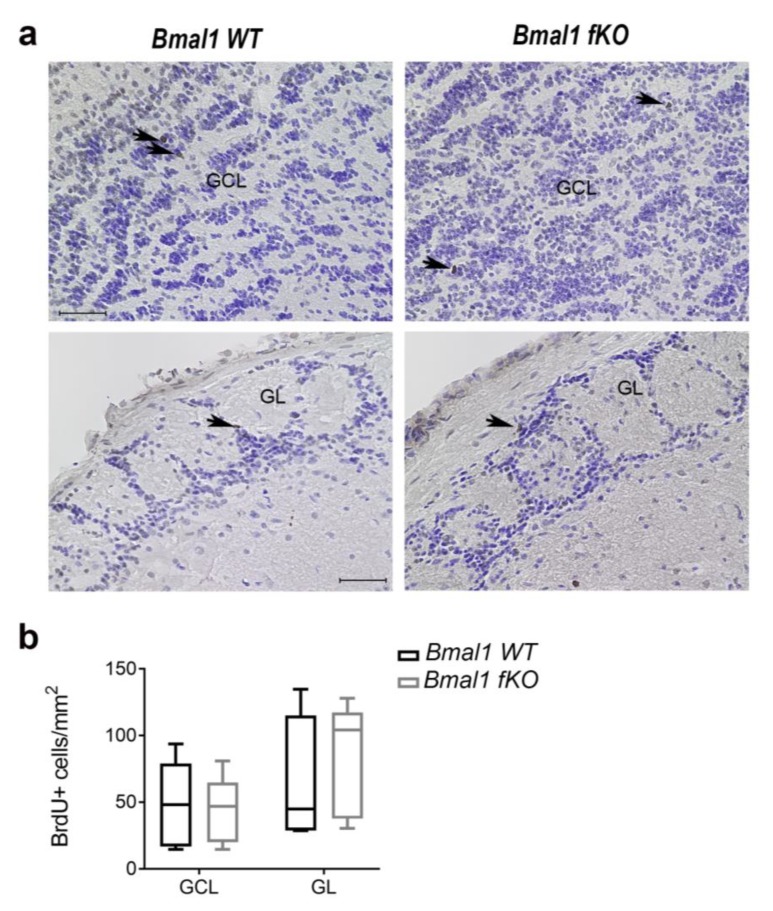
Proliferating cells in the olfactory bulb of *Bmal1 fKO* mice. (**a**) Representative photomicrographs of BrdU+ cells (brown, arrows) and cresyl violet staining (blue) in the granule cell layer (GCL) and the glomerular layer (GL) of the olfactory bulb. Scale bars = 50 µm. (**b**) The number of BrdU+ cells in the GL and the GL of the olfactory bulb was not different between *Bmal1 WT* and *Bmal1 fKO* mice. *n* = 5 mice per genotype.

The number of BrdU+ cells reaching the GCL and the GL of the OB one day after the last BrdU injection was not significantly different between *Bmal1 WT* and *Bmal1 fKO* mice (*p* = 0.8, *n* = 5 per genotype) (Figure 7). Moreover, both the thickness of the RMS, defined by the DCX+ migrating neuroblasts (*p* = 0.8, *n* = 5 per genotype), and the dense network of astrocytes surrounding the RMS, defined by GFAP-IR (*p* = 0.07), were not different between *Bmal1 WT* and *Bmal1 fKO* mice (Figure S1).

Consistently, the cytoarchitecture of the olfactory bulb in cresyl violet stained sections appeared not different between *Bmal1 WT* and *Bmal1 fKO* mice (Figure S2, Table 1).

### 2.6. Forebrain Specific Bmal1 Deletion Affects Reelin Expression in OB

**Figure 8:**
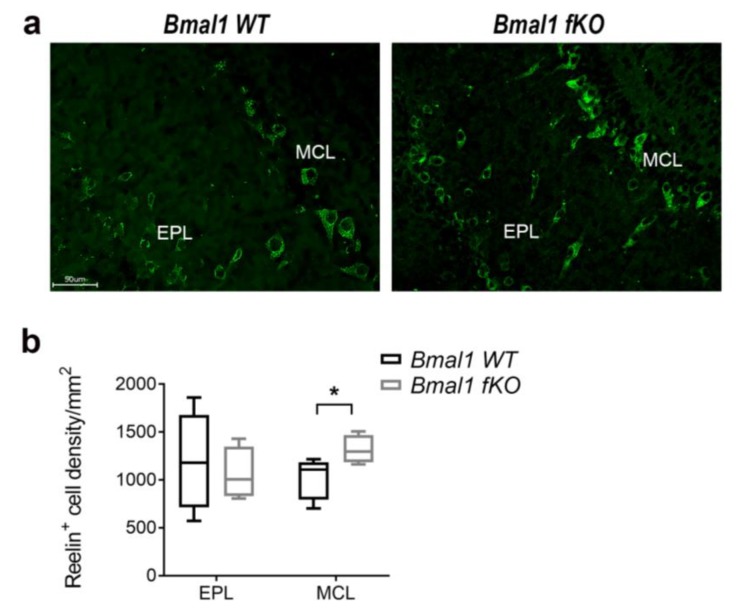
Reelin-immunoreaction in the olfactory bulb of *Bmal1 fKO* mice. (**a**) Representative Reelin-immunoreaction in the external plexiform layer (EPL) and the mitral cell layer (MCL) of the olfactory bulb. Scale bar = 50 µm. (**b**) Quantitative analysis revealed a higher density of Reelin+ cells in MCL (* *p* < 0.05) but not in the EPL of *Bmal1 fKO* (*n* = 4) as compared to *Bmal1 WT* (*n* = 5) mice.

In both genotypes, Reelin+ cells could not be found in the RMS (not shown) but in the mitral cell layer (MCL) and the external plexiform layer (EPL) of the OB (Figure 8). In the EPL, there was no difference in the number of Reelin+ between *Bmal1 WT* (*n* = 5) and *Bmal1 fKO* (*n* = 4) mice (*p* = 0.66) (Figure 8). However, in MCL the number of Reelin+ cells was significantly lower in *Bmal1 WT* (*n* = 5) as compared to *Bmal1 fKO* (*n* = 4) mice (*p* = 0.03) (Figure 8). Interestingly, in the OB of *Bmal1 WT* mice, Reelin-Ir showed a co-localization with Bmal1 (Figure S3).

### 2.7. Olfactory Function Is Not Affected in Forebrain-Specific Bmal1-Deficient Mice

**Figure 9:**
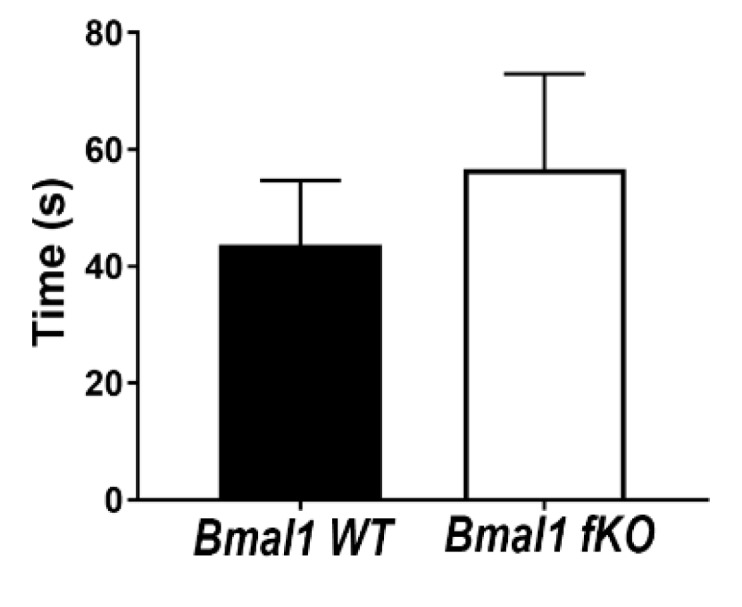
Olfactory function is not affected in *Bmal1 fKO* mice. The latency to find the hidden food was comparable in *Bmal1 WT* and *Bmal1 fKO*. *n* = 14 mice per genotype.

The buried food test was used to test the olfactory function. The latency to find the hidden food was comparable in *Bmal1 WT* (43.7+/−11.0 sec) and *Bmal1 fKO* (56.7+/−16.3 sec) mice (*p* = 0.5, *n* = 14 per genotype) (Figure 9).

## 3. Discussion

In conventional *Bmal1*-deficient (*Bmal1-/-*) mice circadian rhythms and light entrainment of circadian rhythms are impaired [26]. This chronodisruption is associated with increased oxidative stress leading to premature aging, cognitive impairment, impaired adult neurogenesis, and systemic diseases [7,8,9,27]. Mice with a deletion of *Bmal1* in neurons, astrocytes, and oligodendrocytes (*NestinCRE^+^;Bmal1^f/f^)* [25] or excitatory forebrain neurons (*Bmal1 fKO*) [11] show changes in cognitive behavior without the chronodisruption. However, in *NestinCRE^+^;Bmal1^f/f^* mice (Musiek, et al. 2013), these changes in cognitive behavior are associated with a redox defense dysregulation and massive activation of astrocytes, indicative for neuroinflammation. In contrast, in *Bmal1 fKO* mice, there is no activation of astrocytes or generalized increase in oxidative stress, as shown in this study. Thus, the *Bmal1 fKO* mouse is an excellent model to study the role of Bmal1 in the forebrain without chronodisruption and in the absence of hallmarks of neuroinflammation.

Proliferation, differentiation/maturation, migration, and survival of NPCs were not affected in *Bmal1 fKO* mice. Thus, selective deletion of *Bmal1* in Camk2a expressing cells does not affect adult neurogenesis. Moreover, the DG and OB volume and the histological architecture of the OB layers were comparable between *Bmal1WT* and *Bmal1 fKO* mice. Thus, selective deletion of *Bmal1* in Camk2a expressing cells does not affect DG or OB development. This is consistent with the minor role of Camk2a during ontogenetic development [28]. Adult neurogenesis can be affected by modulation of the neuronal circuits on the cellular level, e.g., neuronal activity, [29] or on the systemic level, e.g., locomotor activity and enriched environment [30]. However, this study indicates that the modulation of the forebrain neuronal circuits in *Bmal1 fKO* does not affect adult neurogenesis.

Our previous studies in *Bmal1-/-* mice showed higher oxidative stress in the neurogenic niches of the SGZ and the SVZ/RMS, which was associated with impaired adult neurogenesis [9,10]. However, in *Bmal1 fKO* mice, there was no increase in oxidative stress in the respective neurogenic niches.

Interestingly, in *Bmal1 fKO* mice, oxidative stress was selectively increased in the CA3 region but not in other regions of the hippocampus, although *Bmal1* is present in all hippocampal regions [31]. However, we assume that the astrocytes might cope differently with clearing of reactive oxygen species in the different brain regions. The CA3 region is especially crucial for encoding spatial memory [32], and oxidative stress impairs learning and memory [33]. It has been reported previously that in *Bmal1 fKO* mice spatial memory and learning is impaired, which indicates the involvement of *Bmal1* in the forebrain neuronal circuits apart from its role in the circadian function [11]. The increase in 8-OH(d)G-Ir is indicative of oxidized RNA [10,34], which has been shown to affect signal transmission, neurotransmission, synaptic plasticity, as well as oscillatory networks in the brain [35]. Thus, our data suggest that oxidative stress in the CA3 region might be one factor for the deficits in spatial memory of *Bmal1 fKO* mice shown by Snider and colleagues [11].

In the OB, selectively, the MCL showed higher oxidative stress. Mitral cells are the principal projection neurons in OB. They receive input from the olfactory sensory neurons (OSN), through their apical dendrite [36]. Their lateral dendrites extend over a long distance to form dendrodendritic reciprocal synapses with the inhibitory granule cells. This promotes a lateral inhibition, which is crucial for odor discrimination and for synchronizing odor-induced activities of mitral cells [37]. Furthermore, mitral cells express the extracellular matrix glycoprotein Reelin [24,37]. Reelin plays a critical role in NSCs migration to OB, as it acts as a chemoattractant for the migrating neurons, and is responsible for switching the tangential migration into radial migration, and, subsequently, proper positioning of new-born interneurons in GCL and GL of the OB [38]. Overexpression of Reelin under the control of the Camk2a promotor affects the proliferation of NPCs as well as migration and differentiation of neuroblasts [39]. Interestingly, we observed a higher expression of Reelin in the MCL in Bmal1 fKO mice. However, this increase was not effective to modulate adult neurogenesis.

Besides its role in guiding the NSCs migration, Reelin is involved in neuroplasticity signaling pathways through binding to ApoE receptors. Moreover, Reelin expression is affected by oxidative/neuronal stress resulting in synaptic dysfunction [40,41]. Therefore, we tested whether the olfactory function was affected in *Bmal1 fKO* mice using the buried food test. The latency to find the buried food was not significantly different between *Bmal1 WT* and *Bmal1 fKO* mice. Thus, overexpression of Reelin in the mitral cells of *Bmal1 fKO* has no effect on olfactory function. We did not expect a direct effect of *Bmal1*-deletion from the neurons in the OB itself as conventional *Bmal1*-deficient mice have an intact overall olfactory function [42].

In summary, selective deletion of *Bmal1* in Camk2a expressing cells does not affect adult neurogenesis in the DG or the SVZ/RMS. However, oxidative stress was increased in the CA3 region of *Bmal1 fKO* mice, which might account, at least partially for spatial memory deficits described in the literature. Moreover, Reelin expression was increased in the MCL of *Bmal1 fKO* mice, but this was not associated with a change of neuroblast migration or deficits in olfactory function.

## 4. Material and Methods

### 4.1. Experimental Animals

All animal experiments were approved by the local government, North Rhine-Westphalia State Agency for Nature, Environment and Consumer Protection, Germany (AZ: 84-02.04.2012.A102, 84-02.04.2014.A314) in accordance to international guidelines on the ethical use of experimental animals [43]. All efforts were exerted to decrease the number and suffering of animals.

In Camk2a: Cre transgenic mice (B6.Cg-Tg(Camk2a-cre)T29-1Stl/J, Jackson Laboratory, Bar Harbor, ME, USA) Cre recombinase is expressed under the Camk2a promoter. Camk2a is expressed most abundantly in forebrain mature neurons [44]. This mouse line was crossed with transgenic mice in which *Bmal1* is flanked by loxP sites (B6.129S4(Cg)-Arntl^tm1Weit/J^, Jackson Laboratory, Bar Harbor, ME, USA), allowing recombination between the loxP sites and inactivation of the *Bmal1* gene only in the/cells in which Cre recombinase is expressed [44]. Thus, in the offspring (*Bmal1 fKO*) *Bmal1* is selectively deleted in forebrain mature neurons [11]. The B6.129S4(Cg)-Arntltm1Weit/J mice were used as *Bmal1 WT* mice. Mice were kept for breeding at the local animal facility. Adult male (3–6 months old) mice were used for experiments. Prior to experiments, mice were housed in standard cages in a temperature-controlled environment under 12 h light and 12 h darkness (LD) condition, lights on at 6:00 am. Mice had free access to food and water. Genotype was confirmed by PCR as previously described [11]. Cell type-specific Bmal1 deletion was validated by immunohistochemistry using an anti-Bmal1 antibody (Figure S4). The integrity of the circadian system was validated by spontaneous locomotor activity rhythms in LD and constant darkness (DD) (Figure S5). Consistent with previous findings [11], Bmal1-immunoreaction was reduced in the cerebral cortex (Figure S4a) but not in the SCN (Figure S4b) and the entrainment as well as circadian rhythms of spontaneous locomotor activity were not affected in *Bmal1 fKO* mice (Figure S5).

### 4.2. BrdU Assay

Mice were injected with the S-phase marker BrdU (Roche, Basel, Switzerland) at a dose of 100 mg/kg twice daily, at the beginning and the end of the light phase (ZT2 and ZT12, respectively) for three consecutive days. To study NPCs proliferation, one group of mice (*n* = 5 per genotype) was sacrificed on the next day after the last BrdU administration. To study NPC survival and neuronal differentiation, a second group of mice (*n* = 5 per genotype) was sacrificed 28 days after the last BrdU administration.

### 4.3. Tissue Processing

Animals were deeply anesthetized using Ketamine/Xylazine (100 mg/10 mg/kg body weight, respectively). Mice were perfused with 0.9% NaCl transcardially followed by 4% paraformaldehyde using a Ministar Peristaltic Pump (World Precision Instruments, Sarasota, FL, USA). Brains were removed from the skull, post-fixed in 4% paraformaldehyde for 24 h followed by cryoprotection in 20% then 30% sucrose, each for 24 h. Brains were divided into two sagittal halves. One half was sectioned through the entire rostro-caudal extent of the brain into six 40 μm free-floating coronal parallel sections. The other half was embedded in optimal cutting temperature compound (OCT) and cut into six 20 µm sagittal parallel sections. Sectioning was performed using a cryostat (Leica CM, Wetzlar, Germany).

### 4.4. Immunohistochemistry

After rinsing with phosphate-buffered saline (PBS), sections were incubated with 0.6% H_2_O_2_ for 30 min at room temperature (RT) and then washed in PBS. DNA was denatured using 2N HCl for 30 min at 37 °C, followed by rinsing with 0.1 M boric acid for 10 min at RT. After rinsing with PBS, sections were incubated in 10% normal goat serum in PBS-0.2% Triton for 1h at RT to quench unspecific binding of the secondary antibody. Sections were incubated with rat monoclonal anti-BrdU antibody (1:800, Serotec, oxford, UK), mouse monoclonal anti-8 hydroxy 2′ deoxyguanosine (8-OH(d)G) antibody (1:250, QED Bioscience, San Diego, CA, USA) or rabbit anti-Bmal1 primary antibody (1:1000, generous gift from Prof. D. Weaver) overnight at 4 °C. Sections were incubated with biotinylated goat anti-rat, anti-mouse or anti-rabbit IgG (1:500, Vector Laboratories, Burlingame, CA, USA), respectively, for 1h at RT followed by incubation with VECTASTAIN® Elite® ABC solution (Vector Laboratories, Burlingame, CA, USA) for 1h at RT. Then, sections were rinsed and incubated with 0.05% 3, 3′-Diaminobenzidine (SIGMA-ALDRICH, St. Louis, MO, USA) for 5 min. Sections stained with using an anti-BrdU antibody were counter-stained with Cresyl violet. Staining against Bmal1 was done without DNA denaturation. Sections were mounted and dehydrated. Slides were cover-slipped using Depex (SERVA Electrophoresis, Heidelberg, Germany).

### 4.5. Immunofluorescence

Coronal sections of the hippocampal region were used for double immunofluorescence labeling with BrdU/doublecortin (DCX), or BrdU/NeuN. DNA was denatured as described above. Sections were incubated with a mixture of anti-BrdU (1:500, AbD Serotec, Kidlington, UK) with rabbit polyclonal anti-DCX (1:1000, Abcam, Cambridge, UK) or polyclonal rabbit anti-NeuN (1:1000, Millipore-Chemicon, Burlington, MA, USA). Sections were then rinsed in PBS, followed by incubation with a mixture of the secondary antibodies Alexa Fluor 488 goat anti-rat IgG (1:500, Molecular Probes, Eugene, OR, USA) and Alexa Fluor 568 goat anti-rabbit IgG (1:500, Molecular Probes, Eugene, OR, USA) for 1h at RT. Sections were rinsed, and nuclei were counter-stained with NucBlue (Molecular probes, Eugene, OR, USA). Sections were mounted and dehydrated. Slides were cover-slipped using Vectashield Hard Set anti-fade reagent (Vector Laboratories, Burlingame, CA, USA) and stored in darkness at 4 °C. For staining against Reelin, sagittal sections were incubated with mouse monoclonal anti-Reelin (1:2000, Abcam, Cambridge, UK) and then with Alexa Fluor 488 goat anti-mouse IgG (1:500, Molecular Probes, Eugene, OR, USA) as secondary antibody.

### 4.6. Image Acquisition and Analysis

The animal genotype was obscured to the investigator. The camera settings (exposure time, photo interval, haze reduction condition) were kept identical during images acquisition and processing in all samples. Image acquisition, processing, and analysis of co-localization were performed by BZ-II analyzer software (Keyence Corporation, Osaka, Japan). Immunohistochemically stained sections were analyzed using the bright field mode on a KEYENCE BZ 900E microscope (Keyence Corporation, Osaka, Japan) by a 40X objective.

In one out of six series of coronal sections containing the hippocampus, BrdU-immunopositive (+) cells in the DG were analyzed. The granular cell layer of DG was subdivided into inner, middle and outer thirds while the subgranular zone (SGZ) was considered as a two-nucleus-wide region along the inner border of the granular cell layer of DG towards the hilus. Cells were ranked in regard to their location in the SGZ or granular cell layer of DG and counted manually. The resulting cell number was multiplied by six to estimate the number of BrdU+ cells in the entire hippocampus.

In sagittal sections, BrdU+ cells were analyzed in the SVZ, RMS, and OB. The RMS was divided into two subregions: the vertical limb (VL) and the horizontal limb (HL). The granular cell layer (GCL) and the glomerular layer (GL) of the OB were analyzed separately. Reelin+ cells were analyzed in the mitral cell layer (MCL) and the external plexiform layer (EPL) of the OB. Cells were counted in a delineated area, and the mean cell density in each mouse was expressed as the number of cells/mm^2^.

Mean intensity of 8-OH(d)G-IR in the hippocampus (DG, CA1, CA3), SVZ, RMS (VL, HL), and OB (GCL, GL, MCL) was determined. Quantification of IR was performed using Image J software (available online: http://rsbweb.nih.gov/ij). Background staining in cell-free neuropil was used to define the threshold. The area of positively 8-OH(d)g-IR above the threshold was expressed as the percentage of the total area.

Cytoarchitecture of the OB was determined based on cresyl violet staining; the width of the layers was measured as described previously [45].

Fluorescent signals were analyzed using a 40X objective and respective filters of the KEYENCE BZ 900E (Keyence Corporation, Osaka, Japan). Fifty BrdU+ cells were analyzed for co-localization with DCX, while twenty BrdU+ cells were analyzed for co-localization with NeuN per animal.

For volume analysis, one out of six coronal (DG) and sagittal (OB) series, respectively, was stained with cresyl violet. The boundaries of the DG and the OB were outlined, and the area was multiplied by six and section thickness (40 μm or 20 µm; respectively) to calculate the total volume according to the Cavalieri principle as described before [9]. Volumes were displayed in mm^3^.

### 4.7. Immunoblot

*Bmal1 WT* and *Bmal1 fKO* mice 16 weeks old (*n* = 5 per genotype) were killed with isoflurane. The hippocampus was carefully dissected out under a dissection microscope. Tissue was homogenized and lysed in RIPA buffer (Thermo Scientific, Waltham, MA, USA) with 1% Halt Protease Inhibitor Cocktail (Thermo Scientific, Waltham, MA, USA). Protein concentration was determined using the BCA kit (Thermo Scientific, Waltham, MA, USA).

PAGE and Immunoblot were performed using Novex XCell Sure Lock Electrophoresis and Blot module (Thermo Scientific, Waltham, MA, USA) according to manufacturer’s instructions and Invitrolon™ PVDF membranes (45 µm pores, Life technologies, Carlsbad, CA, USA).

The membranes were washed in TBS-Tween (TBST) and incubated with the blocking solution (TBST containing 5% fat-free milk powder, NEB, Ipswich, MA, USA) for 1 h. Membranes were incubated with rabbit polyclonal anti-GFAP (1:80000, DAKO, Glostrup, Denmark) for 12 h at 10 °C. After washing, membranes were incubated with secondary HRP-conjugated goat anti-rabbit IgG (1:40000, Dianova, Hamburg, Germany) for 1 h. After washing, immunoreactive bands were visualized using Super Signal West ECL (Thermo Scientific, Waltham, MA, USA) by Molecular Imager^®^ ChemiDoc™XRS (BioRad, Hercules, CA, USA). The intensity of the respective immunoreactive bands was normalized against b-Actin using densitometric analyses using ImageJ software.

### 4.8. Behavioral Assays

For analysis of spontaneous locomotor activity, *Bmal1 fKO* (*n* = 5) and *Bmal1 WT* (*n* = 5) mice were individually housed in standard cages with free access to food and water. Spontaneous locomotor activity was recorded using infrared movement detectors linked to a monitoring system (Mouse-E-Motion) (infra.e.motion, Hamburg, Germany). For the analysis of entrainment of spontaneous locomotor activity, the mice were kept in a 12h light/12 h dark cycle (LD) (light on at 06.00, light off at 18.00) for two weeks. For the analysis of a circadian rhythm in spontaneous locomotor activity, the mice were kept under constant darkness (DD) for two weeks. Locomotor activity was measured continuously and recorded in 10 min intervals. For activity profile analysis MatLab R12 based Clocklab software (Actimetrics, Wilmette, IL, USA) was used. Rhythm stability of the period length in LD and in DD was determined by Qp-analysis [6]. The chronotype of mice was determined by the “median of activity” (MoA). The MoA is the time-point on the timescale at which the mouse has achieved 50% of its daily activity. The standard deviation of the MoA (SDevMoA) gives an additional measure of rhythm instability [46].

For assessment of olfactory function in *Bmal1 fKO* and *Bmal1 WT* (*n* = 14 for each genotype), a buried food test was performed as previously described [47,48]. During the habituation period of two weeks, the mice received one hedonistic piece of food (Froot Loops, Kellogg’s, Battle Creek, MI, USA) daily. Before the test phase, the mice were food-deprived for 18 h. The mice were tested individually between 9:00–11:00 am. The mouse was allowed to acclimate for 5 min in the test cage (clean cage filled with a 3-cm depth of bedding), then transferred temporarily into a holding cage. The mouse was transferred again to the test cage after a single Froot Loop was completely hidden 1 cm beneath the surface in one corner of the cage. The latency to find the Froot Loop was recorded as soon as the mouse touched the bedding until it held the Froot Loop with the paws or when the mouse started to eat it. Mice that couldn’t find the food within 300 s were excluded from the analysis (one *Bmal1 fKO* mouse).

### 4.9. Statistical Analysis

Statistical analysis was performed using Graph Pad Prism software. Mann-Whitney-U test, unless stated otherwise, was used to determine differences between groups. *p*-value < 0.05 was considered statistically significant. Values are presented as mean ± SEM.

## 5. Conclusions

We conclude that forebrain specific *Bmal1* deletion leads to an increase in oxidative stress in subregions of the cerebral cortex, such as the CA3 region of the hippocampus and in the mitral cell layer of the olfactory bulb. However, forebrain specific *Bmal1* deletion but does not affect adult neurogenesis in the neurogenic niches, the subgranular zone, and the subventricular zone. Moreover, forebrain *Bmal1* deletion leads to an increase in Reelin expression in the mitral cell layer of the olfactory bulb but does not affect overall olfactory function.

## Figures and Tables

**Table 1:** Volume and cytoarchitecture of the olfactory bulb (OB) in *Bmal1 WT* (*n* = 3) and *Bmal1 fKO* mice (*n* = 3) based on cresyl violet-stained sections. The thickness of the following layers was analyzed separately: external plexiform (EPL), mitral cell (MCL), internal plexiform (IPL), and glomerular layer (GL).

Genotype	Total OB Volume (µm^3^)	GL (µm)	EPL (µm)	MCL (µm)	IPL (µm)
***Bmal1 WT***	10.2 + 0.52	100.3 + 10.98	161.6 + 2.7	29.07 + 1.5	32.08 + 1.5
***Bmal1 fKO***	10.04 + 0.42	118.3 + 19.8	165.5 + 19.6	34.28 + 3.9	35.3 + 4.5
***p* value (*t*-test)**	0.8	0.4	0.8	0.2	0.5

## Supplementary Materials

The supplementary materials are available online at https://www.mdpi.com/1422-0067/21/4/1394/s1.
