# Supplementary figures and images for: Impact of Targeted Deletion of the Circadian Clock Gene Bmal1 in Excitatory Forebrain Neurons on Adult Neurogenesis and Olfactory Function
Source: Int J Mol Sci. 2020 Feb 19;21(4):1394. doi: 10.3390/ijms21041394 (PMC7073072; doi:10.3390/ijms21041394)

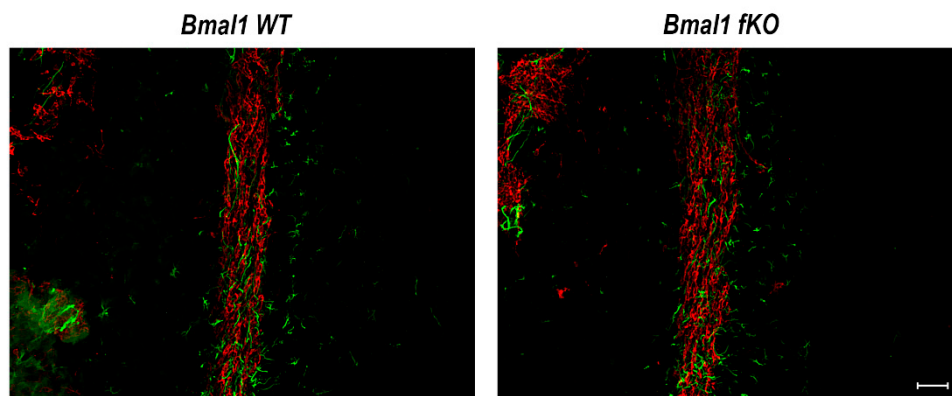

Figure S1

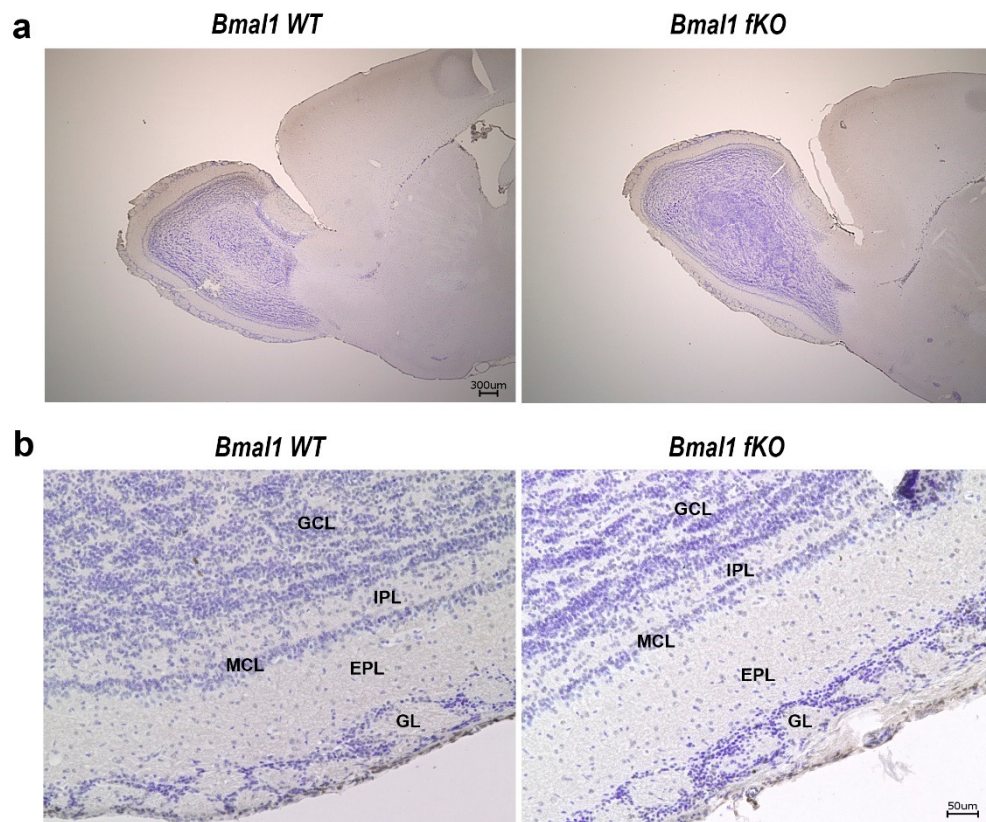

Figure S2

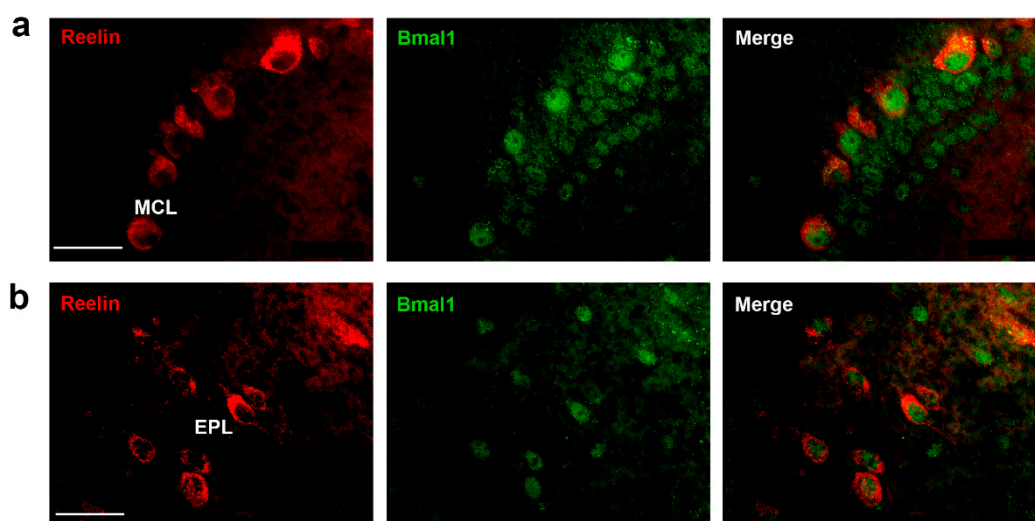

Figure S3

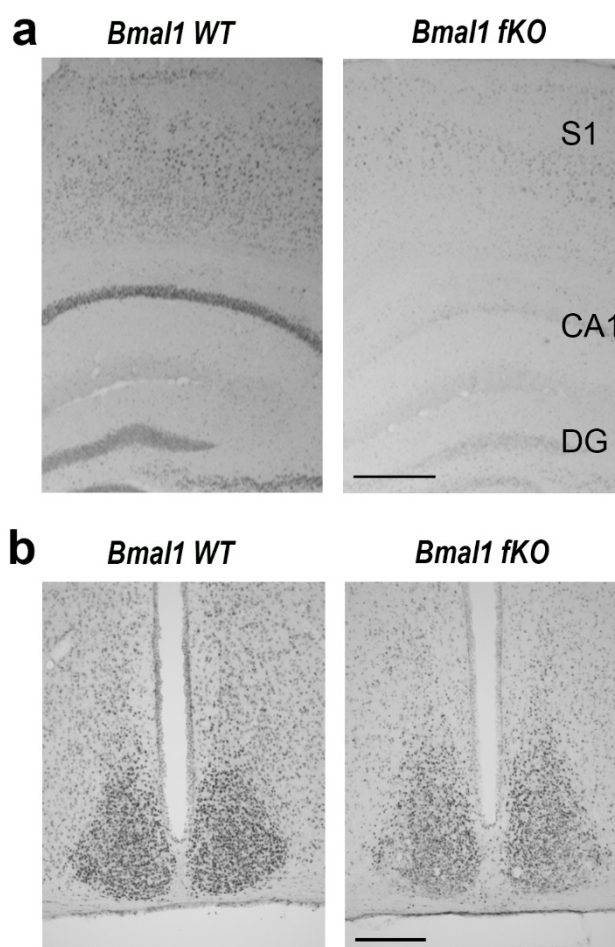

Figure S4

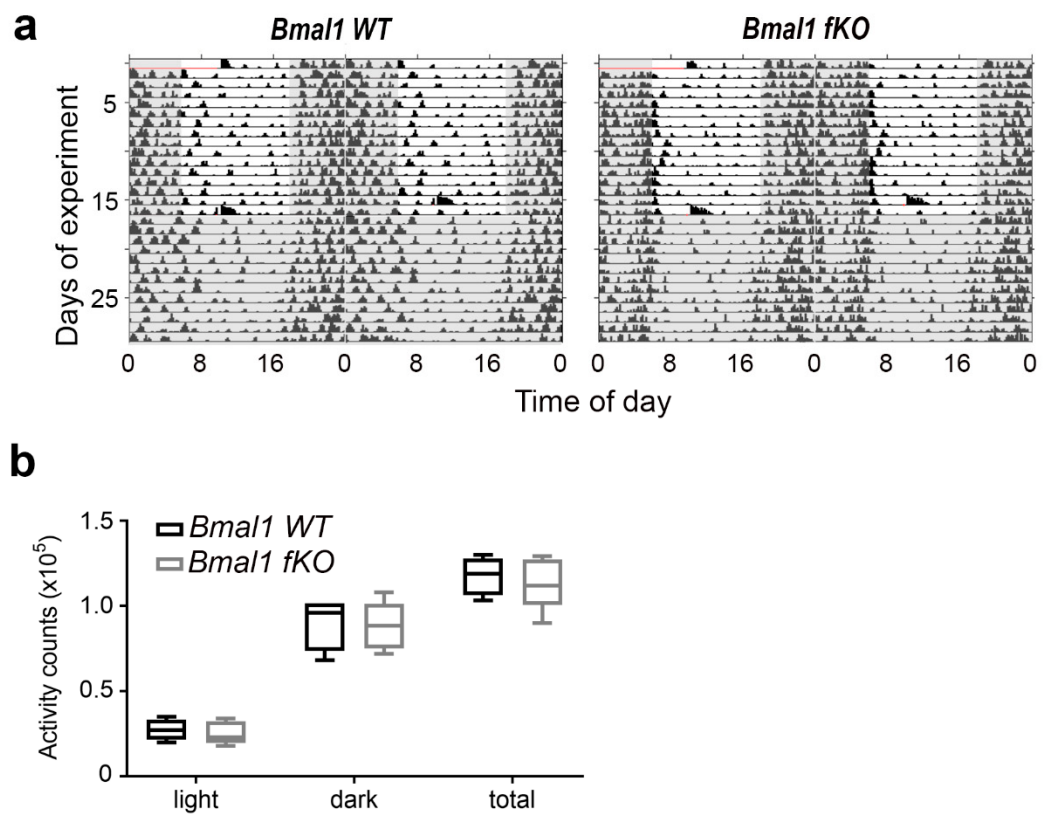

Figure S5

Supplement: Supplementary file 1 [file ijms-21-01394-s001.pdf]
